# Molecular identification and prevalence of tick-borne pathogens in zebu and taurine cattle in North Cameroon

**DOI:** 10.1186/s13071-019-3699-x

**Published:** 2019-09-11

**Authors:** Babette Abanda, Archile Paguem, Mamoudou Abdoulmoumini, Manchang Tanyi Kingsley, Alfons Renz, Albert Eisenbarth

**Affiliations:** 1 Institute of Evolution and Ecology, Department of Comparative Zoology, University of Tübingen, Auf der Morgenstelle 28, 72076 Tübingen, Germany; 2 Programme Onchocercoses field station of the University of Tübingen, P.O. Box 65, Ngaoundéré, Cameroon; 3 School of Veterinary Medicine and Sciences, Department of Parasitology and Parasitological Diseases, University of Ngaoundéré, Ngaoundéré, Cameroon; 4 Department of Biological Sciences, University of Ngaoundéré, P.O. Box 454, Ngaoundéré, Cameroon; 5 Institute of Agricultural Research for Development (IRAD), Wakwa Regional Centre, P.O. Box 65, Ngaoundéré, Cameroon; 6 Present Address: Institute of Novel and Emerging Infectious Diseases, Friedrich-Loeffler-Institut, Südufer 10, 17493 Greifswald-Insel Riems, Germany

**Keywords:** Tick-borne pathogen, Cattle, Cameroon, *Anaplasma*, *Borrelia*, *Ehrlichia*, *Rickettsia*, *Theileria*

## Abstract

**Background:**

Public interest for tick-borne pathogens in cattle livestock is rising due to their veterinary and zoonotic importance. Consequently, correct identification of these potential pathogens is crucial to estimate the level of exposition, the risk and the detrimental impact on livestock and the human population.

**Results:**

Conventional PCR with generic primers was used to identify groups of tick-borne pathogens in cattle breeds from northern Cameroon. The overall prevalence in 1260 blood samples was 89.1%, with 993 (78.8%) positive for *Theileria/Babesia* spp., 959 (76.1%) for *Anaplasma*/*Ehrlichia* spp., 225 (17.9%) for *Borrelia* spp., and 180 (14.3%) for *Rickettsia spp*. Sanger sequencing of a subset of positively-tested samples revealed the presence of *Theileria mutans* (92.2%, 130/141), *T. velifera* (16.3%, 23/141), *Anaplasma centrale* (10.9%, 15/137), *A. marginale* (30.7%, 42/137), *A. platys* (51.1%, 70/137), *Anaplasma* sp. ‘Hadesa’ (10.9%, 15/137), *Ehrlichia ruminantium* (0.7%, 1/137), *E. canis* (0.7%, 1/137), *Borrelia theileri* (91.3%, 42/46)*, Rickettsia africae* (59.4%, 19/32) and *R. felis* (12.5%, 4/32). A high level of both intra- and inter-generic co-infections (76.0%) was observed. To the best of our knowledge, *B. theileri, T. mutans*, *T. velifera*, *A. platys*, *Anaplasma* sp. ‘Hadesa’, *R. felis* and *E. canis* are reported for the first time in cattle from Cameroon, and for *R. felis* it is the first discovery in the cattle host. *Babesia* spp. were not detected by sequencing. The highest number of still identifiable species co-infections was up to four pathogens per genus group. Multifactorial analyses revealed a significant association of infection with *Borrelia theileri* and anemia. Whereas animals of older age had a higher risk of infection, the Gudali cattle had a lower risk compared to the other local breeds.

**Conclusion:**

Co-infections of tick-borne pathogens with an overall high prevalence were found in all five study sites, and were more likely to occur than single infections. Fulani, Namchi and Kapsiki were the most infected breed in general; however, with regions as significant risk factor. A better-adapted approach for tick-borne pathogen identification in co-infected samples is a requirement for epidemiological investigations and tailored control measures.

## Background

Tick-borne pathogens (TBPs) have severely impaired livestock productivity worldwide, with an increasing risk for the human population due to their potential zoonotic character [1]. In tropical Africa, ticks are vectors for a large variety of diseases, such as piroplasmoses caused by the protozoans *Babesia* and *Theileria*, bacterial infections with species of the genera *Anaplasma* (anaplasmosis), *Borrelia* (relapsing fever), *Ehrlichia* (heartwater), *Rickettsia* (spotted fever), and also many viral diseases, like Crimean-Congo hemorrhagic fever [2]. These infectious diseases cause considerable losses and diminish the economic value of livestock where the enzootic status remains unstable [2].

In Cameroon, which is one of the main regional providers of beef and other products derived from cattle, the population is dominated by zebu and crossbreeds (European taurine × zebu and African taurine × zebu), with the taurine cattle population at risk of extinction due to widespread and uncontrolled admixture [3]. The main local vectors for TBPs are hard ticks of the genera *Amblyomma*, *Haemaphysalis*, *Hyalomma* and *Rhipicephalus* [4]. Pure *Bos taurus indicus* cattle have been reported less susceptible to TBPs than pure *Bos taurus taurus* cattle, based on attractiveness for the respective tick vectors and/or due to more effective immunological responses [5].

The prevalence of the various TBPs and their interdependences in Cameroon are not well investigated. Most of the studies used conventional microscopy of blood-smears, serology, or *post-mortem* analyses [6, 7] which all have considerable limitations. Identification of individual species of pathogens is almost impossible without the intervention of molecular tools, like PCR. Moreover, studies on the prevalence of the locally available TBPs in Cameroon and in particular on the level of co-infection is scarce. The present study aims to investigate the occurrence of TBPs in the cattle population, including “mild” and “non-pathogenic” conspecifics and their level of co-infection. Furthermore, the level of exposition and infection of different cattle breeds in Cameroon to TBPs, and the potential risk of exposure for the human population is highlighted.

## Methods

### Study sites and location

**Fig. 1:**
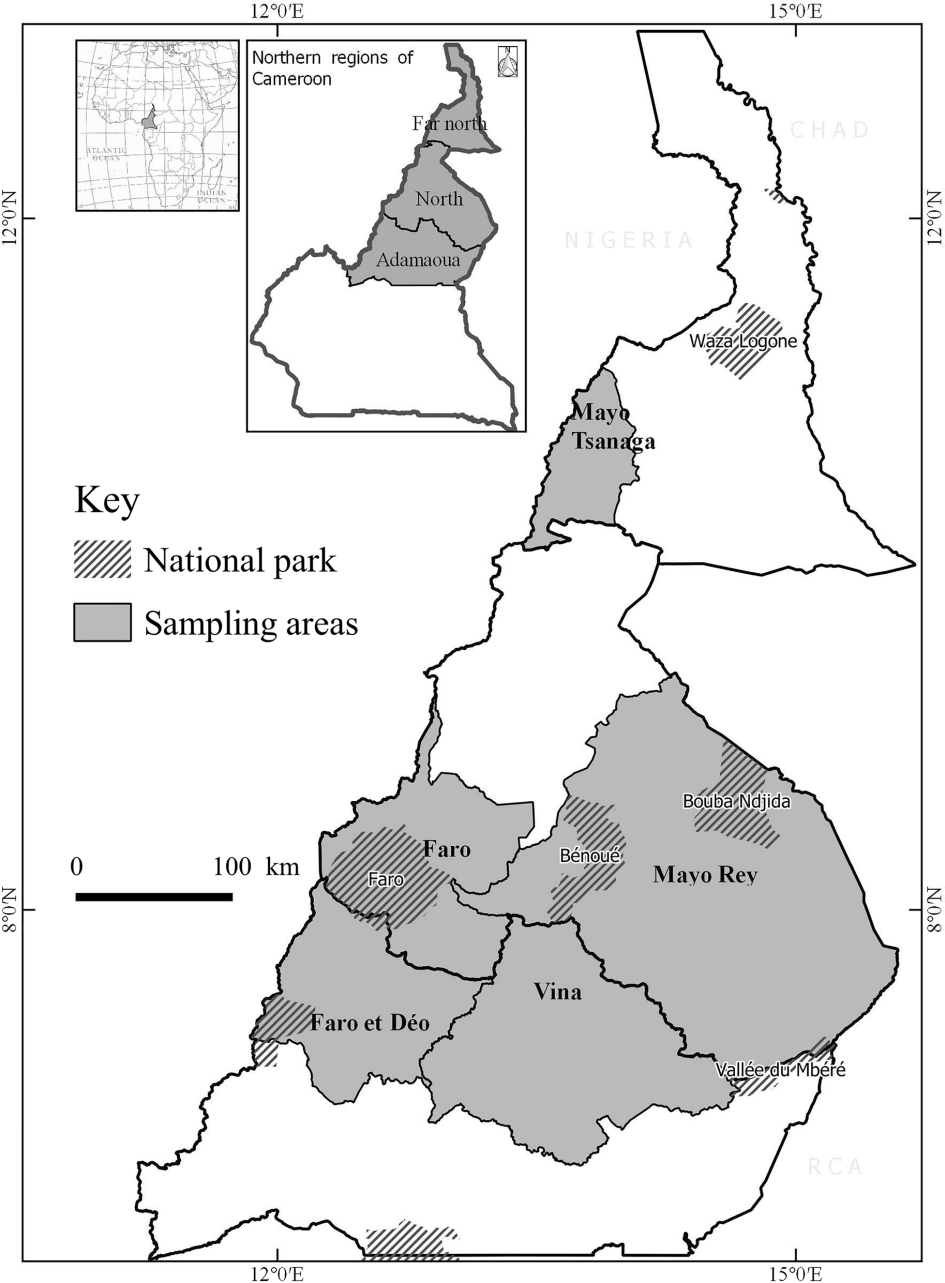
Sampling areas in the northern part of Cameroon. The Vina and Faro et Deo sites are located in the Adamaoua region, the Faro and the Mayo-Rey in the North and the Mayo Tsanaga in the Far North region. The shaded zones represent the sampling areas and the zones with stripes the national parks

The sampling took place from April 2014 (end of the dry season) to June 2015 (middle of the rainy season). A total of 1260 cattle were examined in three different bioclimatic zones in the northern part of Cameroon. The corresponding sites (Fig. 1) were the Adamaoua highlands with 64,000 km^2^ of surface, representing the sub-humid Guinea savannah biotope, the North with 67,000 km^2^, representing the semi-arid Sudan savannah, and the Far North with 34,000 km^2^, representing the arid Sahel region. Sampling time was generally in the morning and mostly during the rainy season (April until October). Five sites were visited in the three regions: Vina (*n* = 396 cattle examined) and Faro et Deo (*n* =198) in the Adamaoua; Faro (*n* = 175) and Mayo-Rey (*n* = 310) in the North; and Mayo Tsanaga (*n* = 181) in the Far North.

### Field work, sampling procedure and DNA isolation

For each herd visited, approximately 10% of the cattle were sampled. Parameters of age in years, sex, breed [Gudali; White and Red Fulani grouped as Fulani; Bokolodji (= Zebu *Bos taurus indicus*); Namchi/Doyao; Kapsiki (*=* autochtonous *Bos taurus taurus*); Charolais (= European *Bos taurus taurus* and cross-breed)], weight and body condition score (BCS) were taken from each animal. The BCS varied from 1 to 5 according to the fat and muscle appearance: 1–2, poor; 3–4, good; and 5, very good (convex look or blocky). The weight was standardized as recommended by Tebug et al. [8] using the formula LW = 4.81 HG–437.52 (where LW is live weight and HG is thoracic girth measurement in cm). The age was assessed by the dentition [9] and by the information of the herd keeper. Sampled animals were grouped as weaners (1–2.5 years-old), adults (2.5–4.5 years-old), old (4.5–8 years-old) and very old (> 8 years-old).

Approximately 5 ml of blood per animal was collected from the jugular vein in 9 ml ethylene diamine tetra acetic acid (EDTA) treated vacutainer tubes (Greiner Bio-One, Frickenhausen, Germany) and analyzed for packed cell volume (PCV) [10]. Briefly, approximatively 70 µl of collected whole blood was transferred into heparinized micro-hematocrit capillaries and centrifuged for 5 min at 12,000× *rpm* in a hematocrit centrifuge (Hawksley & Sons Limited, Lancing, UK). The solid cellular phase in relation to the liquid serum phase was measured using the Hawksley micro hematocrit reader (MRS Scientific, Wickford, UK). A PCV below the threshold level of 26% was considered anemic. The remaining whole blood was centrifuged at 3000× *rpm* for 15 min. Plasma was collected for immunological studies (not applicable here) and the remaining fraction (red blood cells and buffy coat) was used for DNA isolation.

Samples of 300 µl of the erythrocyte and cellular fraction were purified using the Wizard® Genomic DNA Purification Kit (Promega, Madison, USA) according to the manufacturer’s instruction. For sample preservation, 50 µl of trehalose enriched 0.1× Tris EDTA (TE) solution (c = 0.2 M, Sigma-Aldrich, Taufkirchen, Munich, Germany) was added as DNA stabilizing preservative in the tube containing the extracted DNA [11], vortexed and spun down. All samples were stored at room temperature in a dry and light-protected environment after being left to dry at 37 °C. Rehydration was done in the laboratory in Tübingen using 75 µl 0.1× TE buffer at 35 °C for at least 10 min until the pellet was completely resolved, and immediately stored at − 20 °C.

### Polymerase chain reaction for tick-borne pathogens

In 25 µl sample reaction tubes, 12.5 µl of the 2× RedMaster Mix (Genaxxon Bioscience, Ulm, Germany) were mixed with the corresponding primer pairs to the final concentration of 1 pmol/µl. One microliter of template DNA and molecular grade water (Sigma-Aldrich) were added to complete the volume at 25 µl. As a negative control, molecular-grade water (Sigma-Aldrich) was used, and positive controls were kindly shared by colleagues from the Freie Universität Berlin, Germany. For the detection of *Borrelia* spp., 1 µl of the first PCR reaction was used as a template for the second amplification in a nested PCR. The corresponding gene loci, primer pairs and annealing temperatures are shown in Table 1.

**Table 1:** Selected primer pairs and annealing temperature for the detection of mitochondrial target regions for the genera *Babesia*/*Theileria*, *Anaplasma*/*Ehrlichia*, *Rickettsia* and *Borrelia*

Genus	Primer	Target gene	Primer sequence (5′-3′)	Annealing T (°C)	Amplicon size (bp)	References
*Babesia*/*Theileria*	RLB-F2	*18S* rDNA	GACACAGGGAGGTAGTGACAAG	57	460–500	[12]
	RLB-R2		CTAAGAATTTCACCTCTGACAGT			
*Anaplasma*/*Ehrlichia*	AnaEhr16S_f	*16S* rDNA	AGAGTTTGATCMTGGYTCAGAA	55	460–520	This study
	Ana-Ehr16S_r		GAGTTTGCCGGGACTTYTTC			
*Rickettsia*	Rick-F1	*16S* rDNA	GAACGCTATCGGTATGCTTAACACA	64	350–400	[13]
	Rick-F2		CATCACTCACTCGGTATTGCTGGA			
*Borrelia* outer	16S1A	*16S* rDNA	CTAACGCTGGCAGTGCGTCTTAAG	63	1205	[14]
	16S1B		AGCGTCAGTCTTGACCCAGAAGTT			
*Borrelia* inner	16S2A	*16S* rDNA	AGTCAAACGGGATGTAGCAATAC	56	600–720	[14]
	16S2B		GTTATTCTTTCTGATATCAACAG			

*Abbreviation*: T, temperature

The PCR cycling conditions were: initial denaturation at 95 °C for 3 min, denaturation at 95 °C for 30 s and elongation at 72 °C for 30 s repeated 35 times, and final elongation at 72 °C for 10 min (MasterCycler EP S Thermal Cycler®, Eppendorf, Hamburg, Germany). All samples were visualized through electrophoresis on a 1.5% agarose gel stained with Midori Green (Nippon Genetics Europe, Düren, Germany). Selected positive reactions were prepared following manufacturer’s recommendations (Macrogen, Amsterdam, Netherlands) and sent for sequencing. Obtained sequences were compared to the non-redundant database GenBank (NCBI) using BLASTN (http://blast.ncbi.nlm.nih.gov/) in the Geneious 9.1 software (Biomatters, Auckland, New Zealand).

### Phylogenetic tree

Annotated sequences of the same genus and locus were extracted from the GenBank database, and aligned with the MUSCLE algorithm using standard parameters. Maximum Likelihood trees based on the Tamura-Nei model with 1000 bootstrap replications were generated using the software MEGA6 [15]. Initial trees for the heuristic search were obtained by applying the Neighbor-Joining method to a matrix of pairwise distances estimated using the Maximum Composite Likelihood approach. Furthermore, a discrete Gamma distribution was used to model evolutionary rate differences among sites. The rate variation model allowed some sites to be evolutionary invariable. *Babesia bigemina* was selected as the outgroup for the *Theileria* tree, whereas *Wolbachia pipientis* was the outgroup for both *Anaplasma/Ehrlichia* and *Rickettsia* trees.

### Statistical analysis

Descriptive statistics were performed to summarize TBP frequency, percentage, and proportion in study sites and co-infection levels according to region and breed. Multivariate logistic regression (MLG) analysis and descriptive statistics were performed using R v.3.4.2 (www.R-project.org) with the *ISLR* package for the MLG. The association between pathogen acquisition and independent variables were examined by computing the odds ratios (OR), 95% confidence intervals (CI) and *P*-value and using the logit equation in the logistic regression model. Each TBP species was used independently as outcome in separate equations. The other variables (PCV, BCS, age, sex, region and breed) were used as baseline predictors. All cattle breeds with less than 10 sampled individuals and all TBP species with less than 10 infected animals were excluded from the logistic regression. A *P*-value below 0.05 was considered statistically significant.

## Results

### Cattle breeds examined and sampling sites

A total of 1306 cattle were examined in the three administrative regions of North Cameroon (Adamaoua, North, Far North) of which 1260 blood samples were used for molecular analyses. The different categories sex, age group, breed, region, BCS and PCV, together with the population prevalence of TBPs are summarized in Table 2. Data from seven different groups of cattle breed were gathered, including four zebu breeds Gudali (*n* = 687), White/Red Fulani grouped as Fulani (*n* = 116) and Bokolodji (*n* = 6), two indigenous taurine breeds Namchi/Doyao (*n* = 181) and Kapsiki (*n* = 200), cross-breeds (*n* = 37), and Charolais (*n* = 27). Most examined animals were female (76.9%). The age ranged from 1 to 16 years and the PCV from 11 to 55%.

**Table 2:** Prevalence of TBPs per screened genera according to PCR results, sex, packed cell volume, body condition score, cattle breed, age and region

Variable	Category	Total	*Anaplasma*/ *Ehrlichia*	*Borrelia*	*Rickettsia*	*Babesia*/ *Theileria*
	PCR-positive		959/1260	225/1260	180/1260	993/1260
	Sequenced		187/959	46/225	63/180	167/993
	Identified		146/187	42/46	34/63	141/167
Sex	Female		736/959	166/225	139/180	760/993
	Male		223/959	59/225	41/180	233/993
PCV	≤ 25	114/1148	19/114	28/114	17/114	104/114
	≥ 26	1034/1148	107/1034	146/1034	123/1034	793/1034
BCS	1–2	82/1247	18/82	17/82	1/82	69/82
	3–4	1062/1247	111/1062	188/1062	135/1062	847/1062
	5	103/1247	7/103	17/103	15/103	72/103
Breed	Bokolodji	6/6	5/6	2/6	0/6	6/6
	Charolais	24/27	21/27	8/27	5/27	24/27
	Cross-breeds	35/37	29/37	9/37	2/37	35/37
	Fulani	107/109	97/109	22/109	10/109	107/109
	Gudali	480/590	480/590	88/590	103/590	472/590
	Kapsiki	171/180	171/180	54/180	32/180	169/180
	Namchi/Doayo	156/174	131/174	36/174	27/174	156/174
Age group (yrs)	1–2.5	157/175	152/175	48/175	31/175	157/175
	> 2.5–4.5	361/402	359/402	96/402	74/402	361/402
	> 4.5–8	398/462	376/462	68/462	58/462	398/462
	> 8	77/84	72/84	13/84	17/84	77/84
Region	Adamaoua		462/522	123/522	80/522	466/522
	Far North		171/180	54/180	32/180	169/180
	North		326/421	48/421	68/421	358/421

### Prevalence of TBPs by PCR

The blood samples of all 1260 animals were analyzed for TBP detection by conventional PCR with group-specific primer pairs for *Babesia/Theileria* spp., *Anaplasma/Ehrlichia* spp., *Borrelia* spp. and *Rickettsia* spp. The number of PCR-positive cases was 993 (78.8%) for *Babesia/Theileria* spp., 959 (76.1%) for *Anaplasma/Ehrlichia* spp., 225 (17.9%) for *Borrelia* spp., and 180 (14.3%) for *Rickettsia* spp. (Table 2). Nine hundred and three (80.4%, 903/1123) of all infected cattle were found to carry at least two of the screened pathogen groups, and the overall TBP prevalence was 89.1% (1123/1260) with every individual carrying at least one of the groups described above. The Adamaoua region had an overall prevalence of 87.9% (522/594) for all pathogens combined.

### Logistic regression of pathogen acquisition with independent variables

Each of the identified pathogens (*n* = 7) was used as outcome in a logistic regression analysis. The results are reported in Table 3. Logistic regression analyzing the association of all TBPs as outcome to environmental and health factors highlighted the Kapsiki breed and older age as main risk factors (OR: 1.96, CI: 0.8–0.97, *P* = 0.01 and OR: 8.8, CI: 2.0–6.2, *P* = 0.002, respectively).

**Table 3:** Logistic regression model with all independent variables as exposure and their interaction with odds of being infected by the corresponding TBP species. *P-*values below 0.05 and level of significance are shown in bold

TBP	Region	Age	Sex	PCV	BCS	A.cn	A.H	A.mg	A.pl	B.th	R.af	T.mt	T.vl
A.cn OR	1	0.9	8.9	7.4	3.5		2.7	2.4	6.5	3.0	4.7	2.2	1.2
95% CI	− 4.7–0.2	0.6–1.1	0.2–3.9	0.1–3.1	1.7–2.3		na	na	0.02–4.7	0.7–11	na	1.1–5.0	0.9–4.4
*P*	0.07	0.5	0.8	0.6	0.3		0.9	0.9	0.7	0.09	0.2	**0.002** **	**0.002** **
A.H OR	1.0	0.9	0.2	< 0.0001	4.3	2.7		2.3	1	1.3	6.7	8.5	5.6
95% CI	0.007–0.7	0.6–1.4	0.03–0.9	na	0.3–43.0	na		na	0.1–6.1	na	Na	1.8–3.7	6.7–5.5
*P*	**0.04** *	0.8	0.05	0.99	0.2	0.9		0.9	0.9	0.9	0.9	**0.003** **	**0.0001** ***
A.mg OR	3.4	0.9	0.3	1.4	0.4	< 0.0001	< 0.001		0.3	2	0.8	14.8	4.2
95% CI	1.3–9.3	0.7–1.0	0.1–0.9	0.3–4.7	0.05–1.8	na	na		0.02–1.2	0.5–6.7	0.1–4.4	6.4–35.3	0.5–24.1
*P*	**0.009** *	0.3	**0.03** *	0.5	0.3	0.9	0.99		0.15	0.2	0.9	**< 0.0001** ***	0.1
A.pl OR	1.9	0.8	2	0.9	0.3	1.1	1.2	0.2		1.2	0.7	22.4	2.6
95% CI	0.9–3.9	0.7–0.9	0.8–5.2	0.3–2.4	0.08–1.1	0.1–6.1	0.2–6.7	0.05–0.9		0.4–3.3	0.1–3.0	11.6–4.6	0.5–1.1
*P*	0.06	**0.02** *	0.1	0.9	0.1	0.9	0.8	0.05		0.6	0.6	**< 0.0001** ***	0.2
B.th OR	3.5	0.8	1.2	2.9	0.6	2.3	< 0.0001	1.8	1.35		2.1	0.5	1.1
95% CI	2.0–6.2	0.7–0.9	0.8–2.0	1.8–4.6	0.3–1.1	0.5–8.1	na	0.4–5.5	0.4–3.3		0.4–7.6	0.2–1.3	0.2–3.8
*P*	**< 0.0001** ***	**0.003** **	0.3	**< 0.0001** ***	0.1	0.2	0.9	0.3	0.5		0.2	0.2	0.8
R.af OR	1.7	1	0.4	1	1	3.6	< 0.0001	1	1.1	1.9		8.4	2.06
95% CI	0.5–6.0	0.7–1.2	0.1–1.7	0.2–4.3	0.1–4.9	0.1–34.1	na	0.1–5.1	0.2-–.6	0.3–7.1		2.6–27.9	0.08–1.7
*P*	0.3	0.8	0.2	0.9	0.9	0.3	0.9	0.9	0.8	0.3		**0.0002** ***	0.5
T.mt OR	0.8	1	1	0.4	1.5	12.8	9.3	16.4	21.2	0.6	7.9		6.4
95% CI	0.5–1.4	0.9–1.7	0.5–1.9	0.1–1.0	0.7–3.0	2.0–72.9	2.3–37.0	6.9–39.7	11.1–41.6	0.2–1.5	2.3–2.5		1.6–26.8
*P*	0.5	0.2	0.9	0.08	0.2	**0.004** **	**0.001** **	**< 0.0001** ***	**< 0.0001** ***	0.3	**0.0006** **		**0.007** **
T.vl OR	0.5	0.8	0.9	2.6	3.2	12.4	23.9	4	3.1	0.9	2	5.3	
95% CI	0.08–3.1	0.6–1.1	0.2–3.6	0.7–9.9	0.6–1.3	2.1–62.9	2.6–223	0.4–2.5	0.6–1.3	0.1–3.5	0.07–17.9	1.0–26.9	
*P*	0.4	0.3	0.8	0.1	0.1	**0.002** **	**0.004** **	0.1	0.1	0.9	0.5	**0.04** *	

*Abbreviations*: A.cn, *Anaplasma centrale*; A.H, *Anaplasma* sp. ‘Hadesa’; A.mg, *Anaplasma marginale*; A.pl, *Anaplasma platys*; B.th, *Borrelia theileri*; R.af, *Rickettsia africae*; T.mt, *Theileria mutans*; T.vl, *Theileria velifera*; na, not available; OR, odds ratio; CI, confidence interval

### Pathogen identification and co-infections

**Fig. 2:**
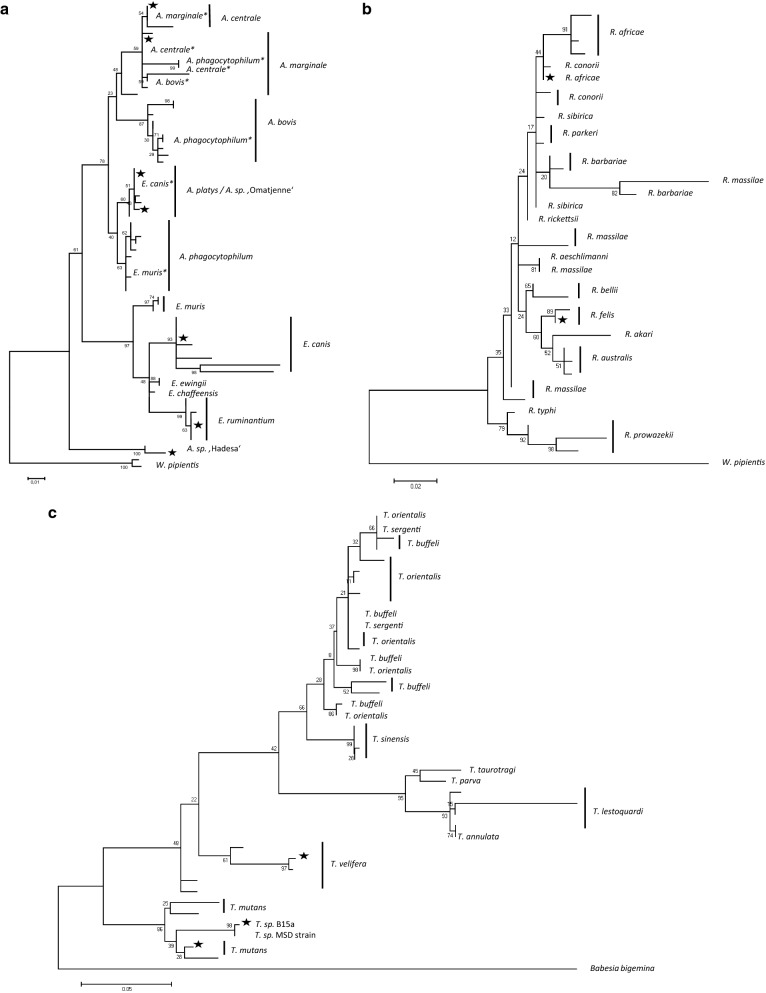
Molecular phylogenetic analysis of selected genera using rDNA markers by Maximum Likelihood method. Evolutionary analyses were conducted in MEGA6. Black stars indicate sequences generated in the present study. Annotations with asterisks indicate likely misidentifications. **a** *Anaplasma*/*Ehrlichia 16S* rDNA dataset (357 positions in final dataset) with *Wolbachia pipientis* as the outgroup. **b** *Rickettsia 16S* rDNA dataset (330 positions in final dataset) with *W. pipientis* as the outgroup. **c** *Theileria 18S* rDNA dataset (394 positions in final dataset) with *Babesia bigemina* as the outgroup

For species identification, 296 of the 1123 PCR positive samples (26.4%) were selected for DNA sequencing, of which 240 (81.0%) could be successfully sequenced. Of these, 78.0% were generated for *Anaplasma*/*Ehrlichia* spp. (146/187), 84.4% for *Babesia*/*Theileria* spp. (141/167), 91.3% for *Borrelia* spp. (42/46), and 53.9% for *Rickettsia* spp. (34/63; Table 2). In total, 12 different species or genotypes were identified by matching with the GenBank database. Ranked after the most prevalent species, these were: *T. mutans*, *A. platys*, *A. marginale*, *B. theileri*, *A. centrale*, *Anaplasma* sp. ‘Hadesa’, *T. velifera*, *R. africae*, *R. felis*, *Theileria* sp. B15a, *E. ruminantium* and *E. canis*. The phylogenetic ML tree compares those genotypes with database entries from GenBank (Fig. 2a–c).

Co-infections with species of the same genus or group of genera were common. The highest percentage of animals with more than three of the five genera of parasites per individual was found in the Far North region (6.1%), followed by Adamaoua (2.8%) and North region (0.8%). The age was significantly associated to the pathogen acquisition (*P* = 0.002) with older animals being more infected. Kapsiki from the Mayo-Tsanaga division were more infected with TBPs (99.4% per region) than Namchi and zebu breeds from other regions (*P* = 0.01).

Single infections were detected in 264 (24.0%) of the 1123 infected cases. Intra-generic double infections that could still be delimitated to the respective species (Table 4), were most frequent for *T. mutans* + *T. velifera* (60.0%), followed by *A. platys* + *A. marginale* (17.3%), and *A. platys* + *Anaplasma* sp. ‘Hadesa’ (9.6%). In 45 cases (52%) of intra-generic co-infections, only one species could be identified. The most common inter-generic combinations were of *T. mutans* + *A. platys*, *T. mutans* + *Anaplasma* sp. ‘Hadesa’, *T. mutans* + *R. africae* and *T. mutans* + *A. marginale*. Gudali breed had less co-infections than Namchi and Kapsiki breeds.

**Table 4:** Proportion of tick-borne pathogens in cattle blood from North Cameroon determined by DNA sequencing

Species	Positive (*n* = 391)	Proportion (%)^a^	Vina (%)^b^	Faro et Deo (%)^b^	Poli (%)^b^	Mayo-Rey (%)^b^	Mayo-Tsanaga (%)^b^
*A. centrale*	15	9.8	2 (13.3)	3 (20.0)	1 (6.7)	1 (6.7)	8 (53.3)
*A. marginale*	42	27.5	6 (14.2)	5 (11.9)	3 (7.1)	21 (50.0)	7 (16.7)
*Anaplasma* sp*. ‘*Hadesa’	15	9.8	0	5 (33.3)	3 (20.0)	7 (46.7)	0
*Anaplasma* sp.	11	7.2	4 (36.4)	3 (27.3)	0	6 (54.5)	0
*A. platys*	70	45.8	20 (28.6)	3 (4.3)	6 (8.6)	33 (47.1)	8 (11.4)
*E. canis*	1	25.0	0	0	0	1 (100)	0
*E. ruminantium*	1	25.0	0	1 (100)	0	0	0
*Ehrlichia* sp.	2	50.0	0	0	0	2 (100)	0
*R. africae*	19	57.6	4 (21.1)	4 (21.1)	1 (5.3)	8 (42.1)	2 10.5)
*R. felis*	4	12.1	0	0	2 (50.0)	1 (25.0)	1 (25.0)
*Rickettsia* sp.	10	30.3	2 (20.0)	3 (30.0)	2 (20.0)	1 (10.0)	2 (25.0)
*B. theileri*	42	100	22 (52.4)	0	2 (4.8)	7 (16.7)	11 (26.2)
*T. mutans*	130	81.8	50 (38.5)	16 (12.3)	9 (6.9)	48 (36.9)	7 (5.4)
*T. velifera*	23	14.5	0	5 (21.7)	5 (21.7)	5 (21.7)	8 (38.1)
*Theileria* sp.	6	3.8	5 (83.3)	0	0	1 (16.7)	0

^a^Proportion of identified species in the respective group of pathogens

^b^Proportion of pathogen-positive samples per site

### Prevalence of *Anaplasma*/*Ehrlichia* species

PCR-positive samples from the *Anaplasma*/*Ehrlichia* group were found mostly in the Vina site on the Adamaoua Plateau (Table 4). Among the 146 positive sequences, 62.0% represented single infections and 38.0% represented co-infections. Single infections of *E. canis* and *E. ruminantium* were found in the sites Mayo Rey and Faro et Deo, respectively (Table 4). According to the proportions of the identified *Anaplasma*/*Ehrlichia* spp. in all study sites the total prevalence was 36.5% for *A. platys*, 21.9% for *A. marginale*, 7.8% for *A. centrale*, 7.8% for *Anaplasma* sp. ‘Hadesa’, 0.5% for *E. ruminantium*, and 0.5% for *E. canis*. Infection with *Anaplasma* spp. increases the likelihood of *Theileria* spp. infection and *vice versa* (Table 3). The age appeared being a risk factor for the acquisition of *A. platys*, with older animals being more infected (OR: 0.8, CI: 0.7–0.9, *P* = 0.02, Table 3).

### Prevalence of *Borrelia* species

*Borrelia* pathogens were identified in all studied regions with the Adamaoua having significantly higher prevalence (OR: 3.5, CI: 2.0–6.2, *P* < 0.0001). The only identified species by sequencing was *B. theileri* with an overall prevalence of 17.9%. Gudali breeds were the least infected cattle with statistical support (*P* = 0.02). Younger animals were significantly less infected (OR: 0.8, CI: 0.7–0.9, *P* = 0.003). *Borrelia theileri* infection was significantly associated to anemia (OR: 2.9, CI: 1.8–4.6, *P* < 0.0001).

### Prevalence of *Rickettsia* species

*Rickettsia* spp. were found in all the regions with no statistical difference. Cattle breed and age was not significantly associated to corresponding infected and non-infected groups. At least one individual from all examined breeds was positive for *Rickettsia* spp., except for Bokolodji (*n* = 6) which was excluded from the logistic regression analysis. The two species identified by sequencing were *R. africae* (prevalence 2.8%) and *R. felis* (prevalence 0.6%). For *R. africae*, the presence of *T. mutans* was a contributing risk factor (OR: 8.4, CI: 2.6–26.9, *P* = 0.0002).

### Prevalence of *Theileria* species

*Theileria mutans* and *T. velifera* were detected in all screened regions. Furthermore, a closely related sequence of *T. mutans*, *Theileria* sp. B15a (GenBank: MN120896) has been detected (Fig. 2c). The overall prevalence of *Theileria* spp. was 57.3% for *T. mutans*, 2.7% for *T. velifera*, 0.5% for *Theileria* sp. B15a and 18.4% for *Theileria* spp. identified only to the genus level. *Theileria mutans* was highly associated with a number of TBP co-infections, including *A. centrale*, *A. marginale*, *A. platys*, *Anaplasma* sp. ‘Hadesa’, *R. africae* and *T. velifera* (Table 3). Furthermore, the taurine breeds, Namchi and Kapsiki were risk factors for *T. velifera* infection (OR: 9.0, CI: 1.4–64.4, *P* = 0.02) and (OR: 7.4, CI: 1.5–42.3, *P* = 0.01) respectively, as well as for co-infections with *A. centrale* and *Anaplasma* sp. ‘Hadesa’ (Table 3).

### Phylogenetic analysis and genetic distances

Maximum Likelihood trees for the genera *Theileria*, *Rickettsia* and *Anaplasma/Ehrlichia* show the evolutionary relationships of the newly acquired sequences in comparison to published GenBank entries (Fig. 2a–c). Most matched very well with published sequences, but also a new genotype in the clade *A. platys*/*Anaplasma* sp. ‘Omatjenne’ (GenBank: MN120891), and another unrecorded genotype closely related to *Anaplasma* sp. ‘Hadesa’ (GenBank: MN124079), were found.

## Discussion

Conventional PCR was used to assess the prevalence of circulating tick-borne parasites and bacteria in cattle from Cameroon’s most important rearing sites in the northern regions. Four different primer pairs targeting ribosomal RNA loci allowed the identification of six genera of important species of TBPs. To the best of our knowledge, our study provides first molecular proof for the presence of *Borrelia theileri*, *Ehrlichia canis*, *Theileria mutans*, *Theileria velifera*, *Anaplasma* sp. ‘Hadesa’, *Anaplasma platys* and *Rickettsia felis* in cattle from Cameroon.

Generally, we found a high TBP prevalence, including a high level of co-infection with other TBP species. Many of the identified TBPs in those cattle are of major economic importance in Africa [16], while some are also causing zoonotic infections in humans. The investigated TBPs differed significantly depending on the cattle breed, age and geographical region, where indigenous taurine breeds, older age and the cattle-rich Adamaoua region were the highest risk factors, respectively. Although the detection and identification of co-infections by using generic primers without cloning can be at times challenging, a sample set of the presently identified species was confirmed by a reverse line blot DNA microarray, albeit with a lower detection rate than the microarray [17].

### *Anaplasma/Ehrlichia* group

*Anaplasma marginale* and *A. centrale* are gram-negative bacteria of the order Rickettsiales, and known to cause bovine anaplasmosis in tropical and subtropical regions [6]. The prevalence in the present study (*A. marginale*: 21.9%, *A. centrale*: 7.8%) was significantly lower than reported in a recent study from North Cameroon with 62.2% and 53.3%, respectively [7], using Giemsa staining. Conversely, our results were higher than reported in the North-West region where the prevalence was 2.2% for *A. marginale* and 0% for *A. centrale*, respectively [6]. The limited mobility of cattle from the ‘Centre de Recherche Zootechnique’ ranch in the North-West region and possibly better husbandry management [6] may explain the lower prevalence and TBP diversity in this area. Moreover, transhumance regularly undertaken by cattle holder in the Adamaoua region could explain the diversity of identified *Anaplasma* species, and the observed prevalence variability [18]. Different study results from the same sampling area in the Vina division are best explained by the alternative technical approaches used for identification. In comparison to molecular tools, microscopic analyses of blood smears are used for rapid diagnostic and informative purposes on the animals’ health status. In fact, identification by microscopy is prone to errors in species identification, as pathogens may look very similar among and between genera leading to misidentification, or may be missed depending on the animals’ patency or developmental status [19]. *Anaplasma marginale* and *A. centrale* are known to be mainly transmitted by ticks of the genus *Rhipicephalus*, in addition to other genera having also been reported as vectors [20]. In Cameroon, *R. appendiculatus* has been identified in the sampling regions as the second most common tick [21], correlating with the high prevalence of these pathogens in the corresponding sites. In our study, sex was significantly associated with the acquisition of *A. marginale*, although with a low odds ratio (OR: 0.3, CI: 0.1–0.9, *P* = 0.03, Table 3).

*Anaplasma* sp. ‘Hadesa’ identified in our sample set had been previously identified in blood samples from Ethiopian zebu cattle [22]. The phylogenetic tree grouped our sequence (GenBank: MN124079) to its clade in a relatively high evolutionary distance from other *Anaplasma* and *Ehrlichia* species (Fig. 2a). In our dataset *Anaplasma* sp. ‘Hadesa’ was inversely correlated with the Adamaoua region, significantly but with low support (OR: 1.0, CI: 0.007–0.7, *P* = 0.04).

*Anaplasma platys* is known as a canine pathogen, causing cyclic thrombocytopenia in dogs. However, it has also been identified in other mammals including cattle, humans and ticks worldwide [23]. In the present study, it was the most commonly detected *Anaplasma* species (prevalence of 36.5%). Two groups of genotypes were found, one of which had yet no listed entry in GenBank (GenBank: MN120882). The absence of detection of this pathogen in previous studies from Cameroon is very likely due to its misidentification for other TBPs [7]. Furthermore, the clade *A. platys* matched very well with *Anaplasma* sp. ‘Omatjenne’ (> 99% identity, GenBank: U54806, Fig. 2a), which was first isolated in sheep and *Hyalomma truncatum* ticks from South Africa [24] and later often diagnosed by its corresponding DNA probes used for reverse line blots assay [25]. In the study by Allsop et al. [24], the complete genome of *Anaplasma* sp. ‘Omatjenne’ (GenBank: U54806) shared 99.9% identity with *Anaplasma* (*Ehrlichia*) *platys* and closely resembled the genome of *E. canis*, most likely due to wrong species annotation [24]. *Rhipicephalus sanguineus* (*sensu lato*) is thought to be the most likely vector of the pathogen which is a tick species already identified in Cameroon [26]. *Anaplasma platys* was identified in 70 specimens of the sequenced subset resulting in a relatively high prevalence (36.5%) in comparison to the records in cattle from Algeria (4.8%) [27], Italy (3.5%) [28] and Tunisia (22.8%) [29]. As a rule, rather than exception, *A. platys* was found in co-infection with other TBPs of the genus *Theileria* with the infection rate increasing with age (Table 3).

*Ehrlichia canis* is a gram-negative bacterium causing canine monocytic ehrlichiosis in dogs and wild canids; these mammals can serve as a natural reservoir for human infections with *R. sanguineus* ticks as a natural vector in tropical and subtropical areas [30]. *Ehrlichia canis* has also been identified in other *Rhipicephalus* species [31]. Among others, the pathogen has been found in dogs from Cameroon [32], Nigeria, South Africa, Portugal, Venezuela [30]. To our knowledge, the present study provides the first evidence for the ocurrence of *E. canis* in cattle from Cameroon. Only one sample from our sequenced subset (*n* = 187) was identified to be *E. canis*. The infected host was a 2-year-old Gudali female cow from the North region in the Mayo Rey site. In fact, cattle paddocks include space for dogs, chicken and other domestic animals living in close proximity. As for most of the TBPs clinically healthy dogs in the subclinical stage can be carriers of *E. canis* for years [33], facilitating the infection of other susceptible hosts. According to the PCV and the BCS, the animal infected by *E. canis* was not suffering from illness albeit co-infected with *T. mutans*. In our study the *E. canis* strain shared 99.6% identity with the *E. canis* amplicon described in Italy and published under the GenBank accession numbers KY559099 and KY559100 [34] (Fig. 2a).

*Ehrlichia* (*Cowdria*) *ruminantium* is the etiological agent of heartwater, also called cowdriosis, in domestic ruminants. The evidence of *E. ruminantium* in Cameroon has been clearly demonstrated in cattle carcasses [6] and the tick vector *Amblyomma variegatum* [35]. Only one positive case of *E. ruminantium* could be identified from our samples subset, representing the second molecular evidence of this pathogen in cattle from Cameroon [36]. The prevalence in our data (0.5%), was significantly lower in comparison to the recently published data (6.6%) on cattle blood from the North and Southwest region of Cameroon [36]. The infected animal was a two years old Red Fulani breed from the Faro et Deo division on the Adamaoua plateau. The BCS was within the range characteristic for an asymptomatic animal, and the PCV level (23 %) indicated anemia. The pathogen was found in co-infections with *A. centrale*, *T. mutans*, *B. theileri* and an unidentified *Rickettsia* sp. The identified strain (GenBank: MN120892) had > 99% sequence identity with the strain ‘Welgevonden’ as previously described from Cameroonian samples [36].

### *Babesia/Theileria* group

*Theileria mutans* and *T. velifera* are known as mild to non-pathogenic species in cattle. *Amblyomma variegatum* ticks transmit *T. mutans*, with the vector being endemic in the northern part of Cameroon. Although age has been reported as a risk factor, our study did not show significant associations (OR: 0.1, CI: 0.9–1.7, *P* = 0.2). *Theileria mutans* is known as non-schizont-transforming of the *Theileria* spp. benign group [37]. However, studies have shown that the presence of the piroplasm at high density in red blood cells can cause disease associated to anemia [38]. The present study did not find any significant difference regarding the PCV level (OR: 0.4, CI: 0.1–1.0, *P* = 0.08). The genotype *Theileria* sp. B15a (GenBank: MN120896) detected, formerly isolated from African buffaloes in South Africa, grouped within the *T. mutans* clade (Fig. 2c) indicating it belongs to the same species.

No schizonts have been described for *T. velifera* [37], whose natural host is the African buffalo, found in high numbers in the Waza National park in the Far North region of Cameroon. This may be the reason for the higher *T. velifera* prevalence in the Kapsiki breed, which are the only cattle kept in this area. No highly pathogenic *Theileria* spp. such as *T. parva* and *T. annulata* was detected in the examined animals. This result indicates either its absence in Cameroon, or the presence below detection levels in cattle formerly or presently infected with *T. mutans* and/or *T. velifera*.

### *Borrelia* group

*Borrelia theileri* is a member of the tick-borne relapsing fever group in contrast to the Lyme borreliosis group [39]. The present study reports for the first time the presence of *B. theileri* in blood samples from cattle in Cameroon. The spirochete bacterium is known to be transmitted to cattle by hard ticks of the genus *Rhipicephalus*, e.g. *R. microplus*, *R. annulatus* and *R. decoloratus* [40]. The pathogen has also been found in *R. geigyi*, however, its capacity as a vector is unknown [40]. Reported cases of tick-borne relapsing fever have been proven responsible for economic losses in livestock [41]. In cattle, *B. theileri* infections have been associated with fever and anemia [41]. In our study area, 17.9% of the studied cattle population was positive for *Borrelia* spp., with *B. theileri* being the only species identified by sequencing.

Furthermore, *B. theileri* was significantly associated with anemia (OR: 2.9, CI: 1.8–4.6, *P* < 0.0001), and present in co-infections with other TBPs in 62% of cases. The highest degree of co-infection comprised *T. velifera*, *T. mutans*, *R. felis*, *A. platys* and *A. centrale.* Similar TBP co-infections excluding *Rickettsia* spp. have been reported [42, 43]. Taurine cattle were significantly more infected than zebu cattle (*P* < 0.01) in line with previously published studies [44], and the difference was significant among age groups with old animals being more infected than their younger counterparts (Table 3). The genotype of *B. theileri* identified in our study (GenBank: MN120889) was 99.9% identical to the strain found in *Rhipicephalus geigyi* from Mali.

### Spotted fever *Rickettsia* group

*Rickettsia africae* is known as the causative agent of African tick bite fever, and has been identified in Cameroon by PCR at a prevalence of 6% from human patients with acute febrile illness without malaria or typhoid fever [35], and at a prevalence of 51% in man from cattle-rearing areas [31]. In previous studies, the pathogen has been identified molecularly in 75% of *A. variegatum* ticks collected from cattle in southern Cameroon [35]. A recent study on ticks collected from cattle in the municipal slaughterhouse of Ngaoundéré in the Adamaoua region in northern Cameroon revealed the presence of *R. africae* among other *Rickettsia* species not identified in our survey [45]. However, the ML tree (Fig. 2b) illustrates the difficulty to clearly distinguish closely related *Rickettsia* spp. when using the *16S* rRNA marker [22]. The genotype of *R. africae* identified in our study (GenBank: MN124096) was 99.7% identical to the strain found in *Hyalomma dromedari* in Egypt and *A. variegatum* in Benin and Nigeria [46].

*Rickettsia felis* is known as an emerging insect-borne rickettsial pathogen and the causative agent of flea-borne spotted fever [47]. Four out of 34 sequenced *Rickettsia* spp. (11.8%) with a prevalence of 0.6% in the sequenced cattle population were detected. The infected animals were from the North region, more precisely from the Faro, Mayo Rey and Mayo-Tsanaga sites, and were in 75% of cases in autochthonous *B. taurus* breeds. The present study reports for the first time *R. felis* in cattle hosts, with previous identification from fecal samples in chimpanzees, gorillas and bonobo apes from Central Africa, including the southern part of Cameroon at a prevalence of 22% [48]. Furthermore, *R. felis* has been identified in *Anopheles gambiae* mosquitoes [49], and human cases were common in Kenya [50] and Senegal [51]. The strain reported in this study (GenBank: MN124093) matches at 99.7% identity with the one described in a booklouse from England as rickettsial endosymbiont (GenBank: DQ652592) and in a cat flea from Mexico [52] indicating they are not predominantly transmitted by ticks, even though they have been found before in tick vectors.

## Conclusions

In North Cameroon, we identified by sequencing of PCR-amplified rDNA from bovine blood at least 11 species of tick-borne pathogens, some of which are known to be pathogenic to livestock or humans alike. *Anaplasma platys*, *Borrelia theileri*, *Ehrlichia canis*, *Rickettsia felis*, *Theileria mutans* and *Theileria velifera* were identified for the first time in cattle from Cameroon. Furthermore, genuinely new genotype sequences related to *A. platys* and *Anaplasma* sp. ‘Hadesa’ were discovered. The high pathogen diversity and levels of co-infection in the livestock population is possibly a result from interaction between different host animals (transhumance or contacts between other domestic and wild animals) and their corresponding tick vectors. In addition to the identification of novel TBP species and genotypes, this study shows the necessity of a universally applicable method for TBP identification unbiased by co-infestations with other related pathogens, which appear in more than 75% of the infected cases.

## Data Availability

The sequences generated during the present study are available in the NCBI GenBank repository under the accession numbers MN120882, MN120888–MN120892, MN120895–MN120896, MN124079, MN124093–MN124096.

## Abbreviations

TBP: tick-borne pathogen
PCR: polymerase chain reaction
PCV: packed cell volume
LW: life weight
GH: thoracic girth
EDTA: ethylene diamine tetra acetic acid
TE: tris-EDTA
NCBI: National Center for Biotechnology Information
BLAST: Basic Local Alignment Search Tool
BCS: body condition score

## References

[1] Dantas-Torres F, Chomel BB, Otranto D (2012). Ticks and tick-borne diseases: a one health perspective. Trends Parasitol.

[2] Wo R, Paweska JT, Petersen N, Grobbelaar AA, Leman PA, Hewson R (2009). Low-density macroarray for rapid detection and identification of Crimean-Congo hemorrhagic fever virus. J Clin Microbiol.

[3] Gauly M, Besbes B, Baker L. Animal genetic resources and their resistance/tolerance to diseases, with special focus on parasitic diseases in ruminants. Joint FAO/INRA workshop Animal genetic resources and their resistance/tolerance to diseases, with special focus on parasitic diseases in ruminants, June 2009, Jouy-en-Josas, France.

[4] Walker AR, Bouattour A, Camicas JL, Estrada-Peña A, Horak IG, Latif AA (2003). Ticks of domestic animals in Africa. A guide to identification of species.

[5] Kashino SS, Resende J, Sacco AMS, Rocha C, Proenca L, Carvalho WA (2005). *Boophilus microplus*: the pattern of bovine immunoglobulin isotype responses to high and low tick infestations. Exp Parasitol.

[6] Ndi C, Bayemi PH, Ekue FN, Tarounga B (1991). Preliminary observations on ticks and tick-borne diseases in the North West province of Cameroon. I. Babesiosis and anaplasmosis. Rev Elev Med Vet Pays Trop.

[7] Abdoulmoumini M, Cyril N, Lendzele S, Kingsley M, Njongui J, Pagnah Z (2017). Bovine babesiosis and anaplasmosis in some cattle farms in the Vina division. Int J Livestock Res.

[8] Tebug SF, Missohou A, Sourokou Sabi S, Juga J, Poole EJ, Tapio M (2016). Using body measurements to estimate live weight of dairy cattle in low-input systems in Senegal. J Appl Anim Res.

[9] Mills PB, Irving JT (1969). Deciduous central incisor tooth development and coronal cementogenesis in cattle. Arch Oral Biol.

[10] McInroy RA (1954). A micro-haematocrit for determining the packed cell volume and haemoglobin concentration on capillary blood. J Clin Pathol.

[11] Jain NK, Roy I (2010). Trehalose and protein stability. Curr Protoc Protein Sci.

[12] Georges K, Loria GR, Riili S, Greco A, Caracappa S, Jongejan F (2001). Detection of haemoparasites in cattle by reverse line blot hybridisation with a note on the distribution of ticks in Sicily. Vet Parasitol.

[13] Nijhof AM, Bodaan C, Postigo M, Nieuwenhuijs H, Opsteegh M, Franssen L (2007). Ticks and associated pathogens collected from domestic animals in the Netherlands. Vector Borne Zoonotic Dis.

[14] Richter D, Matuschka FR (2006). Perpetuation of the lyme disease spirochete *Borrelia lusitaniae* by lizards. Appl Environ Microbiol.

[15] Tamura K, Stecher G, Peterson D, Filipski A, Kumar S (2013). MEGA6: molecular evolutionary genetics analysis version 6.0. Mol Biol Evol.

[16] Lorusso V. Epidemiology and control of cattle ticks and tick-borne infections in central Nigeria. PhD Thesis, University of Edinburgh, Edinburgh, UK; 2015.

[17] Abanda B, Paguem A, Achukwi MD, Renz A, Eisenbarth A (2019). Development of a low-density DNA microarray for detecting tick-borne bacterial and piroplasmid pathogens in African cattle. Trop Med Infect Dis.

[18] Kelly RF, Hamman SM, Morgan KL, Nkongho EF, Ngwa VN, Tanya V (2016). Knowledge of bovine tuberculosis, cattle husbandry and dairy practices amongst pastoralists and small-scale dairy farmers in Cameroon. PLoS ONE.

[19] Salih DA, El Hussein AM, Singla LD (2015). Diagnostic approaches for tick borne haemoparasitic diseases in livestock. J Vet Med Anim Health.

[20] Wesonga FD, Gachohi JM, Kitala PM, Gathuma JM, Njenga MJ (2017). Seroprevalence of *Anaplasma marginale* and *Babesia bigemina* infections and associated risk factors in Machakos county, Kenya. Trop Anim Health Prod.

[21] Tawah CL (1992). Comparative study of tick burdens in Gudali and Wakwa cattle under natural infestation in the subhumid highlands of Wakwa, Cameroon. Preliminary observations. Rev Elev Med Vet Pays Trop.

[22] Hailemariam Z, Krücken J, Baumann M, Ahmed JS, Clausen PH, Nijhof AM (2017). Molecular detection of tick-borne pathogens in cattle from southwestern Ethiopia. PLoS ONE.

[23] Maggi RG, Mascarelli PE, Havenga LN, Naidoo V, Breitschwerdt EB (2013). Co-infection with *Anaplasma platys*, *Bartonella henselae* and *Candidatus Mycoplasma haematoparvum* in a veterinarian. Parasit Vectors.

[24] Allsopp MTEP, Visser ES, Du Plessis JL, Vogel SW, Allsopp BA (1997). Different organisms associated with heartwater as shown by analysis of *16S* ribosomal RNA gene sequences. Vet Parasitol.

[25] Mtshali MS, Steyn HC, Mtshali PS, Mbati PA, Kocan KM, Latif A (2013). The detection and characterization of multiple tick-borne pathogens in cattle at Ficksburg and Reitz (Free State Province, South Africa) using reverse line blot hybridization. Afr J Microbiol Res.

[26] Ndip LM, Ndip RN, Ndive VE, Awuh JA, Walker DH, McBride JW (2007). *Ehrlichia* species in *Rhipicephalus sanguineus* ticks in Cameroon. Vector Borne Zoonotic Dis.

[27] Elelu N, Ferrolho J, Couto J, Domingos A, Eisler MC (2016). Molecular diagnosis of the tick-borne pathogen *Anaplasma marginale* in cattle blood samples from Nigeria using qPCR. Exp Appl Acarol.

[28] Zobba R, Anfossi AG, Parpaglia MLP, Dore GM, Chessa B, Spezzigu A (2014). Molecular investigation and phylogeny of *Anaplasma* spp. in Mediterranean ruminants reveal the presence of neutrophil-tropic strains closely related to *A. platys*. Appl Environ Microbiol.

[29] Said MB, Belkahia H, El Mabrouk N, Saidani M, Alberti A, Zobba R (2017). *Anaplasma platys*-like strains in ruminants from Tunisia. Infect Genet Evol.

[30] Malik MI, Qamar M, Ain Q, Hussain MF, Dahmani M, Ayaz M (2018). Molecular detection of *Ehrlichia canis* in dogs from three districts in Punjab (Pakistan). Vet Med Sci.

[31] Ndip L, Ndip R, Walker D, McBride J (2012). Human ehrlichioses and rickettsioses in Cameroon. Curr Top Trop Med.

[32] Obi TU, Anosa VO (1980). Haematological studies on domestic animals in Nigeria. IV. Clinico-haematological features of bovine trypanosomiasis, theileriosis, anaplasmosis, eperythrozoonosis and helminthiasis. Zentralbl Veterinarmed B.

[33] Stich RW, Rikihisa Y, Ewing SA, Needham GR, Grover DL, Jittapalapong S (2002). Detection of *Ehrlichia canis* in canine carrier blood and in individual experimentally infected ticks with a p30-based PCR assay. J Clin Microbiol.

[34] Santoro M, D’alessio N, Cerrone A, Lucibelli MG, Borriello G, Aloise G (2017). The Eurasian otter (*Lutra lutra*) as a potential host for rickettsial pathogens in southern Italy. PLoS ONE.

[35] Ndip LM, Fokam EB, Bouyer DH, Ndip RN, Titanji VPK, Walker DH (2004). Detection of *Rickettsia africae* in patients and ticks along the coastal region of Cameroon. Am J Trop Med Hyg.

[36] Esemu SN, Ndip RN, Ndip LM (2018). Detection of *Ehrlichia ruminantium* infection in cattle in Cameroon. BMC Res Notes.

[37] Abdela N, Bekele T (2016). Bovine theileriosis and its control: a review. Adv Biol Res.

[38] Mans BJ, Pienaar R, Latif AA (2015). A review of *Theileria* diagnostics and epidemiology. Int J Parasitol Parasites Wildl.

[39] Socolovschi C, Mediannikov O, Raoult D, Parola P (2009). Update on tick—borne bacterial diseases in Europe. Parasite.

[40] McCoy BN, Maïga O, Schwan TG (2014). Detection of *Borrelia theileri* in *Rhipicephalus geigyi* from Mali. Ticks Tick Borne Dis.

[41] Sharma SP, Amanfu W, Losho TC (2000). Bovine borreliosis in Botswana. Onderstepoort J Vet Res.

[42] Wilhelmsson P, Fryland L, Lindblom P, Sjowall J, Ahlm C, Berglund J (2016). A prospective study on the incidence of *Borrelia burgdorferi* sensu lato infection after a tick bite in Sweden and on the Aland Islands, Finland (2008–2009). Ticks Tick Borne Dis.

[43] Uilenberg G, Hinaidy HK, Perié NM, Feenstra T (1988). *Borrelia* infections of ruminants in Europe. Vet Q.

[44] Mattioli RC, Pandey VS, Murray M, Fitzpatrick JL (2000). Immunogenetic influences on tick resistance in African cattle with particular reference to trypanotolerant N’Dama (*Bos taurus*) and trypanosusceptible Gobra zebu (*Bos indicus*) cattle. Acta Trop.

[45] Vanegas A, Keller C, Krüger A, Manchang TK, Hagen RM, Frickmann H (2018). Molecular detection of spotted fever group rickettsiae in ticks from Cameroon. Ticks Tick Borne Dis.

[46] Loftis AD, Reeves WK, Szumlas DE, Abbassy MM, Helmy IM, Moriarity JR (2006). Rickettsial agents in Egyptian ticks collected from domestic animals. Exp Appl Acarol.

[47] Pérez-Osorio CE, Zavala-Velázquez JE, León JJA, Zavala-Castro JE (2008). *Rickettsia felis* as emergent global threat for humans. Emerg Infect Dis.

[48] Keita AK, Socolovschi C, Ahuka-Mundeke S, Ratmanov P, Butel C, Ayouba A (2013). Molecular evidence for the presence of *Rickettsia felis* in the feces of wild-living African apes. PLoS ONE.

[49] Socolovschi C, Pages F, Ndiath MO, Ratmanov P, Raoult D (2012). Rickettsia species in African Anopheles mosquitoes. PLoS ONE.

[50] Richards AL, Jiang J, Omulo S, Dare R, Abdirahman K, Ali A (2010). Human infection with *Rickettsia felis*. Kenya. Emerg Infect Dis.

[51] Socolovschi C, Mediannikov O, Sokhna C, Tall A, Diatta G, Bassene H (2010). *Rickettsia felis*- associated uneruptive fever. Senegal. Emerg Infect Dis.

[52] Nugnes F, Gebiola M, Monti MM, Gualtieri L, Giorgini M, Wang J (2015). Genetic diversity of the invasive gall wasp *Leptocybe invasa* (Hymenoptera: Eulophidae) and of its *Rickettsia* endosymbiont, and associated sex-ratio differences. PLoS ONE.

